# Optimizing Training Population Size and Genotyping Strategy for Genomic Prediction Using Association Study Results and Pedigree Information. A Case of Study in Advanced Wheat Breeding Lines

**DOI:** 10.1371/journal.pone.0169606

**Published:** 2017-01-12

**Authors:** Fabio Cericola, Ahmed Jahoor, Jihad Orabi, Jeppe R. Andersen, Luc L. Janss, Just Jensen

**Affiliations:** 1 Center for Quantitative Genetics and Genomics, Department of Molecular Biology and Genetics, Aarhus University, Tjele, Denmark; 2 Department of Plant Breeding, The Swedish University of Agricultural Sciences, Uppsala, Sweden; 3 Nordic Seed A/S, Odder, Denmark; USDA-ARS Southern Regional Research Center, UNITED STATES

## Abstract

Wheat breeding programs generate a large amount of variation which cannot be completely explored because of limited phenotyping throughput. Genomic prediction (GP) has been proposed as a new tool which provides breeding values estimations without the need of phenotyping all the material produced but only a subset of it named training population (TP). However, genotyping of all the accessions under analysis is needed and, therefore, optimizing TP dimension and genotyping strategy is pivotal to implement GP in commercial breeding schemes. Here, we explored the optimum TP size and we integrated pedigree records and genome wide association studies (GWAS) results to optimize the genotyping strategy. A total of 988 advanced wheat breeding lines were genotyped with the Illumina 15K SNPs wheat chip and phenotyped across several years and locations for yield, lodging, and starch content. Cross-validation using the largest possible TP size and all the SNPs available after editing (~11k), yielded predictive abilities (r_GP_) ranging between 0.5–0.6. In order to explore the Training population size, r_GP_ were computed using progressively smaller TP. These exercises showed that TP of around 700 lines were enough to yield the highest observed r_GP_. Moreover, r_GP_ were calculated by randomly reducing the SNPs number. This showed that around 1K markers were enough to reach the highest observed r_GP_. GWAS was used to identify markers associated with the traits analyzed. A GWAS-based selection of SNPs resulted in increased r_GP_ when compared with random selection and few hundreds SNPs were sufficient to obtain the highest observed r_GP_. For each of these scenarios, advantages of adding the pedigree information were shown. Our results indicate that moderate TP sizes were enough to yield high r_GP_ and that pedigree information and GWAS results can be used to greatly optimize the genotyping strategy.

## Introduction

Standard wheat breeding programs are based on initial cross-pollination between parental lines to produce F_1_ hybrids which, in turn, are self-pollinated to generate segregating F_2_ populations. Subsequently, rounds of self-pollination are performed to restore homozygosity [1]. Until high level of inbreeding is reached, each round of self-pollinations generates thousands of new different individuals increasing the overall phenotypic variation. This variation is explored by breeders which test the new individuals and try to select the ones with the highest performances. However, as a consequence of phenotyping high cost and limited throughput, only a fraction of the potentially very high number of individuals can be tested and enter the selection process.

In the last decades, the development in genomics tools has drastically increased the use of molecular markers, which were predicted to reshape breeding programs especially by reducing phenotyping limitations. One of the main ideas was that, instead of using phenotyping trials, selection could be achieved indirectly using molecular markers that are closely linked to quantitative trait loci (QTLs) responsible of the phenotype [2]. The usage of this selection strategy, named marker assisted selection (MAS), has continued increasing becoming a common practice in commercial crops improvement programs [2,3]. However, the MAS, has proved to be efficient for simple mono/oligo genic traits for which the identification of few markers/QTLs associations with large contributions to phenotypic variation is possible [4,5].

One premises for MAS success was to find markers linked to the QTLs. In the last three decades, an enormous effort was spent on gene mapping both using family-based QTLs mapping [6] and genome wide association mapping (GWAS) [7]. Arguably, the most important conclusion from these studies was that a large part of the genetic variation in commercially relevant phenotypic traits (such as yield, pathogen tolerance, grain quality, and nutrient usage efficiency) was controlled by a constellation of many genes each with small effects and, generally, few genes with large and detectable effects [3]. Although, most of the genes showed a limited contribution to the total genetic variation, often their combined effect proved to be greater than the few genes with large effects [8]. Most mapping studies only had statistical power to detect QTLs with large effects, and genes with minor effects were hard to detect or completely missed [9]. The limitation in linking the larger part of underling QTLs to markers had largely hampered the usage of traditional MAS for complex quantitative traits.

Pedigree-based estimation of breeding values have long been used for genetic improvement of complex traits [10,11]. In 2001, Meuwissen et al.[12] proposed to expand this model to integrate genomic information introducing the concept of genomic prediction (GP). In GP, the combined effects of a large numbers of markers are estimated in a set of individuals that have been genotyped and phenotyped (called training population, TP). The marker effects can be used to calculate the genomic estimated breeding values (GEBVs) of non-phenotyped individuals (referred as breeding population, BP) based on their molecular markers profile. Rather than identifying few markers/QTLs associations, GP jointly analyze all markers attempting to explain the total genetic variance through summing all marker effects irrespective of effect size [12], thus increases our ability to predict poly-genetic traits. The selection strategy based on GP is referred as genomic selection (GS) and has been widely adopted in animal breeding, especially in dairy cattle [13]. GS also showed great potential in improving the genetic gain in crops breeding by reducing the duration of each breeding cycle and/or by increasing the numbers of candidates that can be characterized for a given cost, thus increasing the selection intensity [14]. Several studies have proven that GP would allow an accurate selection in wheat [15–17]. However, in order to efficiently implement GS in wheat breeding programs further investigations are needed on commercial wheat lines.

Firstly the size of the TP, which is the only set of individual that strictly need phenotypic scores, should be optimized. This means that the minimum number of TP individuals that maximize the GP accuracy should be defined in order to keep the need for phenotyping at a minimum. It was reported that the accuracy of the genomic prediction increase when the TP size was increased [18]. However, the gain on GP accuracies was more relevant at small TP size and tends to reach a plateau as TP size increased [19]. Therefore, an optimum TP size can be defined, which balances prediction accuracy in relation to cost of genotyping and phenotyping.

Moreover, the number of individuals available for selection should be maximized to increase selection intensity. This can be done by including extra individuals in the BP, which only needs genotyping. Given a fixed budget for genotyping, the most straightforward way to increase the number of individuals under analysis is to reduce the number of SNPs scored for each individual. Several genotyping platforms offer the possibility to reduce the genotyping throughput to reduce the cost per individual. The uniplex SNPs genotyping platforms are generally conceived for applications requiring small to moderate numbers of SNPs for a large number of samples [20]. TaqMan^®^ (http://www.appliedbiosystems.com) and competitive allele-specific PCR (KASP^®^, http://www.lgcgenomics.com) are two of the most popular techniques available on the market. New generation sequencing technology, such as restriction-site associated DNA sequencing (RAD), are generally used to yield high markers coverage. However, assays can be designed to largely reduce the genome representation (*e*.*g*. by using rare cutting restriction enzyme) and increase the sample multiplexing to reduce the cost per sample [21,22]. Decreasing the number of markers scored reduces the genome coverage resulting in fewer markers in LD with causative mutations and ultimately reducing the accuracy of the predictions. The loss in predictive ability associated with the marker reduction depends on several factors including: i) the genetic architecture of the trait under analysis [23]; ii) the amount of LD in the population under study [18]; iii) the selection strategies used for reducing the markers which can be either random or based on previous information such as association with QTLs [24].

In the present paper, a large set of advanced commercial wheat lines, phenotyped for yield, lodging and starch content, were analyzed with the following aims:

1. Estimate the variant components underling the traits under study and test the accuracies of the genomic predictions;
2. Define the optimal size of the training population;
3. Identify sets of genetic markers associated with QTLs by means of a GWAS;
4. Define the optimal marker set size by quantifying the loss in accuracy of the prediction when: i) the SNPs number was randomly reduced; ii) the SNPs number was reduced based on their GWAS-estimated genomic effects;
5. For these different scenarios, compare the prediction accuracies yielded by pedigree records, genomic data and the combination of the two.

## Material and Methods

### Experimental data

The plant material consisted of 988 F_6_ lines resulting from standard breeding program procedures for *T*. *aestivum* L., at Nordic Seed A/S. Data from three breeding cycles, each consisting around 330 new F_6_ lines, were evaluated. Approximately 60 parental lines were crossed in the beginning of each breeding cycle, generations of selfing followed to produce F_6_ lines. Lines of each breeding cycle were tested for two consecutive years. Cycle1 in 2013–2014, cycle 2 in 2014–2015, cycle 3 in 2015–2016. For the last cycle only 2015 data were available for the present study. For each year, data were collected in three locations in Denmark: Holeby (South DK), Dyngby (Central DK), and Skive (North DK). Line plots of 8.25 m^2^ were organized in trials (42 plots per trial) and each line had two replicates within the trials. For each trial the experimental conditions were highly homogeneous (e.g. sowing time, soil type, application of treatments, assessment time).

The following traits were considered in this paper:

1. Yield (Y): measured as kg/m^2^
2. Lodging (L): assessed by visual evaluation on a 1–9 scale with 1 indicating erect plants 9 indicating plants bending completely to the ground.
3. Starch Content (SC): Evaluate after harvest by means of Near-Infrared Transmission (NIT). The instrument used operates in the near-visible range of 850–1,050 nm, using a monochromator. Last-squares regression was used to compute calibration and composition parameter (for more details see: Williams, 1991 [25]).

Table 1 shows the number of plots recorded and the descriptive statistics (mean, standard deviation, minimum, and maximum) for each trait. Differences in the number of plots are due to missing values.

**Table 1:** Descriptive statistics for the traits considered.

Trait	N of plots	Average	Variance	Min Value	Max value
Yield	10,223	8.93	0.61	3.85	11.5
Lodging	8,751	3.66	4.08	1	9
Starch	10,117	68.76	3.50	60	74

Number of non-missing plots, mean, variance, minimum and maximum values for the three traits considered.

For each line, pedigree records were available up to the grand-parent lines or further back.

Lines were genotyped by Trait Genetics for 15K SNPs using the Infinium wheat SNPs array platform of Illumina. A total of 11,022 SNPs remained for analysis after removal of SNPs i) fixed in the population; ii) with a minimum allele frequency (MAF) lower than 0.05; iii) with more than 10% of missing values. Of these markers 9290 had known map position and 1732 SNPs were unmapped.

### Variance component

A pedigree-based relationship matrix (**A**) was calculated using the tabular method assuming that each parental line had undergone nine cycles of selfing. A genomic relationship matrix (**G**) was computed using the first method described by Van Raden[26]. Firstly, allele-frequencies were arranged in a matrix **M** (*n*×*j*), with *n* indexing the samples and *j* indexing the markers. The matrix **P** contain allele frequencies with column *j* expressed as **1**2(*p*_*j*_ − 0.5) where *p*_*j*_ is the frequency of the second allele at locus *m* and **1** is a vector of ones. The centered matrix **Z** was than obtained by subtracting **P** from **M**. Genomic relationship matrix G were obtained as follow:

$$G=ZZ\prime /2\sum p_{j}(1-p_{j})$$

The phenotypic data were analyzed by Restricted Maximum Likelihood (REML) using the Average Information (AI-REML) module algorithm in the DMU multivariate mixed model package [27]. The raw phenotypic data was analyzed using four different models differing for the terms describing the genetic variance:

$$Model\;I:\;y=Xt+Z_{1}i+Z_{2}f+Z_{3}u+e$$ (1)

$$Model\;AI:\;y=Xt+Z_{4}a+Z_{1}i+Z_{2}f+Z_{3}u+e$$ (2)

$$Model\;GI:\;y=Xt+{Z_{5}g+Z}_{1}i+Z_{2}f+Z_{3}u+e$$ (3)

$$Model\;GAI:\;y=Xt+Z_{5}g+Z_{4}a+Z_{1}i+Z_{2}f+Z_{3}u+e$$ (4)

where ***y*** is the vector of observations; **X** is a design matrix for the fixed factor; ***t*** is the vector of fixed trial effects nested within location, year and breeding cycle; **Z**_n_ are design matrices of random factors; ***g*** is a vector of genomic breeding values for the lines with $g∼N\;(0,\;G\sigma_{g}^{2})$, with $\sigma_{g}^{2}$ being genomic variance and **G** is the genomic relationship matrix; ***a*** is a vector of genomic breeding values for the lines with $a∼N\;(0,\;A\sigma_{a}^{2})$, with $\sigma_{a}^{2}$ being the genomic variance and **A** is the pedigree-based relationship matrix; ***i*** is a vector of line effects with $i∼N(0,\;I\sigma_{i}^{2})$, where $\sigma_{i}^{2}$ is the variance due to uncorrelated lines effects; ***f*** is a vector of G × E interactions (year × location × lines) with $f∼N(0,\;I\sigma_{f}^{2})$; ***u*** is a vector of column spatial effect for each field in each year with $u∼N(0,\;I\sigma_{u}^{2})$ (row spatial effects were tested but excluded from the model because not significant), and ***e*** is a vector of random residuals with $e∼\;N(0,\;I\sigma_{e}^{2})$.

The total phenotypic variance $\sigma_{p}^{2}$ for the model GAI (*Eq 4*) was obtained as:

$$\sigma_{p}^{2}{=\sigma }_{i}^{2}+d(G)\sigma_{g}^{2}+d(G)\sigma_{a}^{2}+\sigma_{f}^{2}/v+\sigma_{u}^{2}/o+\sigma_{e}^{2}/o$$ (5)

where *d(****G****)* is the average diagonal element of the genomic relationship matrix and *d(****A****)* is the average diagonal element of the pedigree-based relationship matrix, *v* is the average number of location by year observed (Y:4.3, L:3.9, SC:4.3) for each genotype, and *o* is the average number of observation (Y:8.5, L:7.16, SC:8.4) for each genotype.

Narrow-sense, heritabilities and broad-sense heritability for model GAI (*Eq 4*) were calculated respectively as:

$$h_{G+A}^{2}=\left(d(G)\sigma_{g}^{2}+d(G)\sigma_{a}^{2}\right)/\sigma_{p}^{2}$$ (6)

$$H_{G+A+I}^{2}=\left(d(G)\sigma_{g}^{2}+d(G)\sigma_{a}^{2}+\sigma_{i}^{2}\right)/\sigma_{p}^{2}$$ (7)

The total explained variance and the heritabilities for the other models were derived with analogous method by removing the terms not present in the equations. For model I, only the broad-sense heritability was calculated.

### Linkage disequilibrium and genome wide association study

The *r*^*2*^ measure [28] was used to compute the LD between markers on the same chromosome and across chromosomes. A decay plot showing *r*^*2*^ between mapped SNPs was used to assess the extent of the LD due to physical linkage within chromosomes.

To identify associations between markers and QTLs linear mixed models were applied. The model used to estimate the effect of the *j*^*th*^ SNPs can be expressed as:

$$y=Xt+m_{j}\beta_{j}+Z_{1}g+Z_{2}f+Z_{3}u+e$$ (8)

where ***y*** is the vector of observations; **X** is a design matrix for the fixed factor; **Z**_n_ are design matrices of random factors; the element ***t***, ***f*, *u*** and ***e*** are defined as in *Eq* 3; ***m***_***j***_ is the vector of marker genotypes at the *j*^*th*^ SNPs; *β* is the *j*^*th*^ SNPs effect; ***g*** is a vector of genomic breeding values for the lines with $g∼N\;(0,\;G_{{-Ch}_{j}}\sigma_{g}^{2})$ with $\sigma_{g}^{2}$ being the genomic variance and $G_{{-Ch}_{j}}$ is the genomic relationship matrix calculated after excluding all the SNPs on the same chromosome as the *j*^*th*^ SNPs and all the un-mapped SNPs. Including $G_{{-Ch}_{j}}$ in the model allowed *i*) to limit the spurious association due to the population structure [7]; *ii*) to avoid underestimation of the *j*^*th*^ SNPs association by adding its effect or the effect of the other marker in LD with it into the relationship matrix. The model was run for each SNP separately.

The genetic variance explained by each locus was computed as follows (Falconer and Mackay 1996):

$$\alpha_{j}=2p_{j}(1-p_{j})\beta_{j}^{2}$$ (9)

where *p*_*j*_ is the MAF of the *j*^*th*^ SNPs and *β*_*j*_ its estimated effect.

### Genomic prediction accuracy

Genomic predictions were carried out by BLUP using three different models: AI (*Eq 2*), GI (*Eq 3*) and GAI (*Eq 4*). The accuracies of the genomic predictions (r_GP_) were calculated as correlation between the phenotypic values corrected for the fixed effect and the estimated breeding values (EBV). Before computing r_GP_, the corrected phenotype was averaged across replications for each line, hence r_GP_ represent the accuracy in predicting line performances when own performance is not included in the model. The EBVs were *a*, *g*, and *a* + *g* for the models AI, GI, and GAI respectively. Different cross validation (CV) strategies were chosen to address different scenarios.

To assess the accuracy yielded by the largest possible TP and all the available genomic information, a leave one out cross validation (LOO-CV) was performed. In this scenario, only one line per time was excluded by the TP and predicted using the data from all other lines (*i*.*e*. the prediction models were run once for each line). The r_GP_ was then computed as the correlation between corrected phenotypes and predicted breeding values.

Subsequently, the effect of the training population size on the r_GP_ was explored. In this scenario, CV with reduced TP sizes ranging from 5% (49 lines) to the 95% (939 lines) of the whole panel were used by randomly sampling the lines to be included in TP. For each TP size, 50 replicates were used. For each of the models under analysis, t-tests were used to assess whether r_GP_ observed at different TP size were significantly different from the estimate yielded by the LOO-CV (when the largest TP was adopted). The t-test used in this contest was expected to be a very conservative test, in order to drive conclusion about these results we suggest considering the trends of the plotted value together with the significances levels.

Additional CV schemes were used to explore the effect of reducing the number of SNPs on the r_GP_.

First, the effect on r_GP,_ when the number of SNPs was reduced using a semi-random selection, was tested. In this scenario, SNPs were randomly sampled but equalizing their distribution across the genome. The following procedure was used: *i*) Given the total number of SNPs to sample, define the number of SNPs to sample for each chromosome to be proportionated to: SNP_*Chr*_/*SNPs*_*TOT*_; *ii*) for chromosome *n* (with *n* = [1, 2, …, 21]), random sample a SNP; *iii*) exclude all the SNPs positioned ± *d* from the already selected SNP (initial *d* = 10 cM); *iv*) if there are remaining SNPs to sample for the chromosome *n* and not all SNPs have already been selected or excluded move to step *ii*; *v*) if there are still SNPs to sample for the chromosome *n* but all SNPs have already been selected or excluded than *d = d/2* and move to step *iii*; *vi*) if there are no more SNPs to sample for the chromosome *n* than *n* = *n*+1 and move to step *ii*. The un-mapped SNPs were included as well in the SNPs sets, in a number defined according to step *i*, but using a completely random selection.

This sampling procedure was used to define SNPs sets ranging from 30 to 10K. Each SNPs set was replicated 50 times and used to calculate **G**. Subsequently, all G matrices were tested 20 times using CV, in which the TP was randomly sampled to be 80% of the total lines. Both model GI and GAI were considered. Standard t-tests were used to assess whether r_GP_ observed at different SNPs set size were significantly different from the r_GP_ estimate obtained when using all the SNPs.

Finally, the effect on r_GP_ was tested, when the number of SNPs was selected based on GWAS result. In order to use the GWAS-defined markers sets in a CV test, the predicted individual cannot be included in the panel used for the association study [29]. To avoid this, for each trait 50 GWAS were performed on the 80% of the total population, the remaining 20% was used to calculate r_GP_ in the CV. For each of the GWAS, SNP sets, ranging from 30 to 10K, were defined according with the amount genetic variance explained (*α*, defined in Eq 9) by each marker. In order to limit the inclusion of markers in the same region the following SNPs selection procedure was adopted: *i*) select the SNP with the highest α; *ii*) exclude all the SNPs positioned ± *d* from the selected SNP (initial *d* = 10 cM); *iii*) if there are still SNPs to select for and not all SNPs have already been selected or excluded move to step *i*; *v*) if there are still SNPs to select but all SNPs have already been selected or excluded than *d = d/2* and move to step *ii*. Following this procedure, only one marker per pick of associated SNPs was selected (until the number of SNPs to select approximates the total number of total SNPs). S2 Fig displays an example of this selection procedure.

Each SNP set was used to calculate **G.** Subsequently, each **G** was tested 50 times using CV in which the TS were the same used to perform the GWAS that defined the SNPs set. Both models GI and GAI were considered.

Other than comparing r_GP_ observed at different SNPs set size with the one observed, when using all the SNPs, t-tests were also used to assess whether the SNPs sets selection based on GWAS yielded higher r_GP,_ when compared with the semi-random selection.

## Results

### Variance components

A significant amount of phenotypic variance was observed for the three traits (Table 1).

Four models were used to estimate the variance components with different factors describing the genetic variance (*Eqs* 1–4). Variance components together with broad sense and narrow sense heritability estimates are reported in Table 2. Note that both $\sigma_{g}^{2}$ and $\sigma_{a}^{2}$ describe the additive genetic variance according to estimated relationships between lines, $\sigma_{g}^{2}$ by using genomic information (**G** used as variance/covariance matrix), $\sigma_{a}^{2}$ as described by the pedigree (**A** used as variance/covariance matrix). The uncorrelated line variance $\sigma_{i}^{2}$ was included to capture the genetic variance that is not traced by the relationship matrices (an identity matrix **I** was used as variance/covariance matrix for this term).

**Table 2:** Traits estimated variance components and predictive abilities.

Trait	Model	$\sigma_{g}^{2}$	$\sigma_{a}^{2}$	$\sigma_{i}^{2}$	$\sigma_{f}^{2}$	$\sigma_{u}^{2}$	$\sigma_{e}^{2}$	h^2^	H^2^	r_GP_
Y	I	-	-	0.09 (0.010)	0.14 (0.004)	0.03 (0.002)	0.09 (0.002)		0.66	-
	GI	0.08 (0.012)	-	0.03 (0.005)	0.14 (0.004)	0.03 (0.002)	0.09 (0.002)	0.51	0.70	0.52
	AI	-	0.12 (0.023)	0.00 (0.013)	0.14 (0.004)	0.03 (0.002)	0.09 (0.002)	0.72	0.72	0.48
	GAI	0.06 (0.011)	0.05 (0.017)	0.00 (0.010)	0.14 (0.004)	0.03 (0.002)	0.09 (0.002)	0.71	0.70	0.55
L	I	-	-	1.69 (0.150)	0.6 (0.028)	0.03 (0.007)	0.70 (0.017)		0.87	-
	GI	1.64 (0.223)	-	0.62 (0.070)	0.6 (0.028)	0.03 (0.007)	0.70 (0.017)	0.65	0.90	0.56
	AI	-	1.72 (0.394)	0.44 (0.223)	0.61 (0.028)	0.03 (0.007)	0.70 (0.017)	0.71	0.89	0.43
	GAI	1.55 (0.222)	0.24 (0.163)	0.49 (0.112)	0.6 (0.028)	0.03 (0.007)	0.70 (0.017)	0.71	0.90	0.56
SC	I	-	-	0.31 (0.022)	0.21 (0.016)	0.42 (0.028)	0.63 (0.014)		0.64	-
	GI	0.33 (0.044)	-	0.07 (0.015)	0.21 (0.016)	0.42 (0.028)	0.63 (0.014)	0.57	0.70	0.57
	AI	-	0.43 (0.090)	0.00 (0.051)	0.21 (0.016)	0.43 (0.028)	0.63 (0.014)	0.71	0.71	0.42
	GAI	0.28 (0.042)	0.12 (0.048)	0.00 (0.029)	0.21 (0.016)	0.42 (0.028)	0.63 (0.014)	0.70	0.70	0.58

The narrow sense heritability (h^2^) broad sense heritability (H^2^) and predictive abilities (r_GP_) using all the markers and in a LOO-CV are also reported.

For all the models used, the traits showed significant genetic variance. Model I, where no relationship matrices were included, estimated H^2^ were 0.66, 0.87 and 0.64 for Y, L and SC, respectively.

Using models including factors accounting for variance described by genomic relationship (model GI, AI or GAI), result in an increased estimation of the broad sense heritabilities for all three traits.

In model GI, a large part of the genetic variance was traced by **G**, the observe h^2^ were 0.51, 0.65 and 0.57 for Y, L and SC, respectively. However, a high fraction of residual genetic variance ($\sigma_{i}^{2}$) was still observed for this model. On the other hand in model AI, **A** was capable to trace all the genetic variance for both Y and SC and a larger part of the genetic variance of L, when compared to **G**. This resulted in increased h^2^ when using model AI which was 0.72, 0.71 and 0.71 for Y, L and SC, respectively. Using model GAI did not result in an increase of the estimate total genetic variance or of the additive genetic variance. In this model, the fraction of the additive genetic variance explained by **G** was similar (Y) or higher than the one explained by **A** (L, SC).

### Linkage disequilibrium and genome wide association study

Results from the LD analysis are displayed in S1 Fig. When considering the whole genome, the LD showed to have a rather long extent within chromosomes with an overall average r^2^ of 0.05, 2.7% of marker with r^2^ > 0.5 and an average distance of the markers with r^2^ >0.5 of 8.64 cM. Differences in the LD patterns were observed for the different chromosome sets. For the genome A, B and D, the average r^2^ was 0.05, 0.05 and 0.11, the percentage of marker with r^2^ > 0.5 was 2.6%, 2.3% and 11.3%, and the average distance of markers with r^2^ >0.5 was 9.47 cM, 8.38 cM and 7.73 cM respectively. Weaker LD was observed across chromosome, with an average r^2^ of 0.01 and 0.1% of the markers with r^2^ > 0.5.

**Fig 1:**
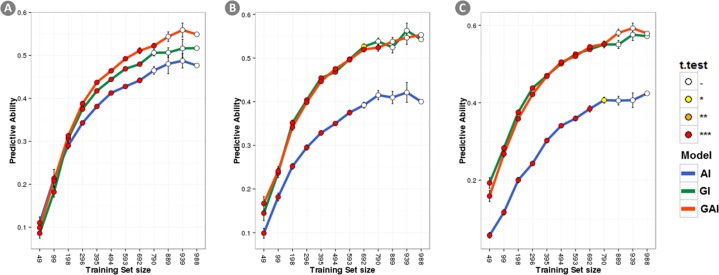
Manhattan plots. GWAS results for the three traits under analysis are displayed: a) Yield; b) Lodging; c) Starch content.

The Manhattan plots displaying the GWAS result are shown in Fig 1. As a consequence of the extensive LD groups of neighboring SNPs are often associated with the same QTL. Table 3 shows the marker with the highest genomic effect for each QTL region detected. The complete list of significant markers (at p-value < 0.05) is reported in S1 Table.

**Table 3:** SNPs showing significant associations with the three traits considered.

Trait	Marker	p-value	Genomic effect	Chr
Y	NOS_wheat_1A_218	2.78E-07	0.0029	1A
Y	NOS_wheat_UNMAP_9781	1.75E-06	0.0028	Un-mapped
Y	NOS_wheat_UNMAP_10354	1.38E-06	0.0025	Un-mapped
L	NOS_wheat_UNMAP_10072	9.36E-09	0.1058	Un-mapped
L	NOS_wheat_3A_3259	7.22E-11	0.0847	3A
L	NOS_wheat_5A_5582	1.19E-06	0.0299	5A
SC	NOS_wheat_3B_4116	2.74E-11	0.0170	3B
SC	NOS_wheat_UNMAP_10138	3.29E-06	0.0156	Un-mapped
SC	NOS_wheat_1B_732	1.43E-06	0.0083	1B

Only the marker explaining the highest genomic effect among all the neighboring (± 5cM) significant ones is reported. A table showing details of all the significant markers is reported in the supplementary materials (S1 Table).

For yield, 12 SNPs showed significant association and one QTL was identified on the chromosome 1A. In this region, the SNP with the largest effect described 2.63% of total genomic variance. Two more significant, un-mapped markers were identified describing 2.54% and 2.27% of the total genomic variance respectively.

For lodging, 13 SNPs showed significant association and two QTLs regions were identified mapping on chromosome 3A and on chromosome 5A describing 3.71% and 1.31% of the genomic variance, respectively. Additionally, one un-mapped SNP was significant describing 4.46% of the genomic variance.

For Starch content 27 significant SNPs showed significant association and two QTLs regions were identified mapping on chromosome 1B and on chromosome 3B explaining 2.07% and 4.25% of the genomic variance, respectively. One additional un-mapped SNP was significant describing 3.75% of the genomic variance.

## Genomic prediction

Table 2 reports the observed r_GP_ when the LOO method was used for CV. In this scenario, all the SNPs were used and the training population was the largest possible. For model AI, using **A** as relationship matrix, the observed r_GP_ were 0.48, 0.43 and 0.42 for Y, L and SC, respectively. The model GI, using **G** as relationship matrix, always outperforms the model AI yielding r_GP_ of 0.52, 0.56 and 0.57 for Y, L and SC. When compared to model GI, model GAI using both **G** and **A** gave higher (for yield) or similar (lodging, starch content) r_GP_.

**Fig 2:**
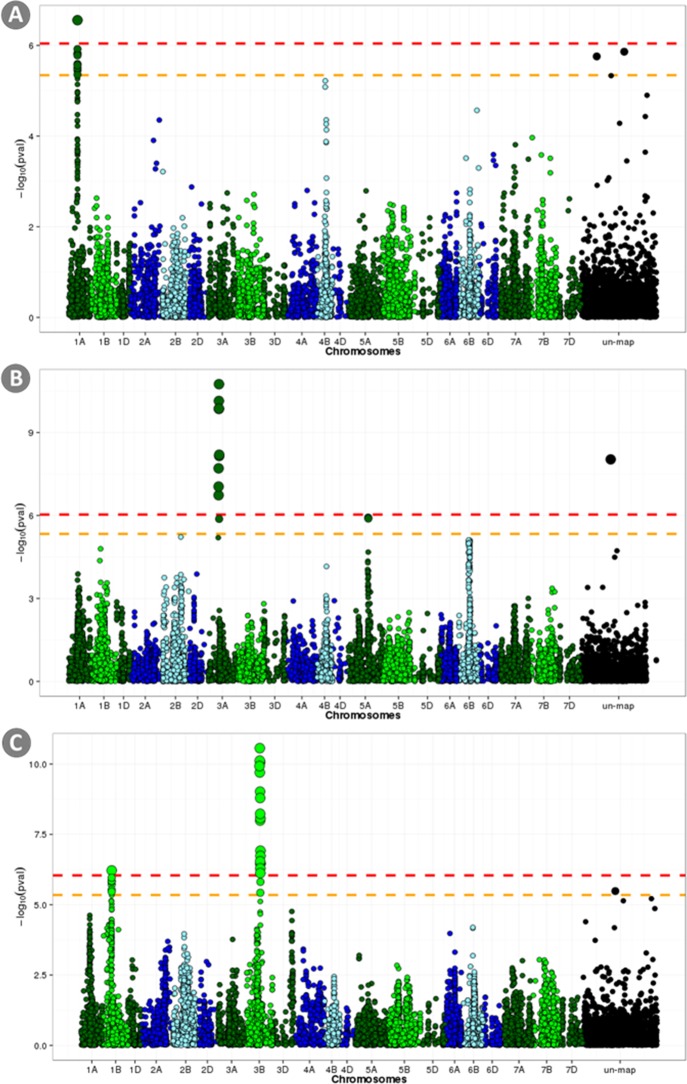
Prediction accuracy as a function of the training set size. Results are displayed for the three traits under analysis: a) Yield; b) Lodging; c) Starch content. Three models were considered: a) A+I in blue; b) G+I in green; c) A+G+I in red. The color of the dots show if each r_GP_ was significantly lower than the highest observed r_GP_ obtained with each model.

Fig 2 shows r_GP_ with progressively reduced TP size. Low but significantly higher than zero r_GP_ were obtained even when as few as 49 random lines (5% of the total number of lines) were used as TP. A progressive increase of the r_GP_ was observed for all the traits when the TP size was increased. However, the observed increase became progressively less with increasing TP size and it plateaued before reaching the maximum TP size. Between 700 and 800 lines were enough to reach the highest r_GP_ observable for all traits (slightly depending on the trait under analysis and the model used). The trend in r_GP_ loss observed when using the **G** matrix was not mitigated by adding the **A** matrix and for very low TP size no difference was observable between GI and GAI models. When compared to the other models, model AI, only using the **A** matrix showed worse r_GP_ even at very low TP size.

**Fig 3:**
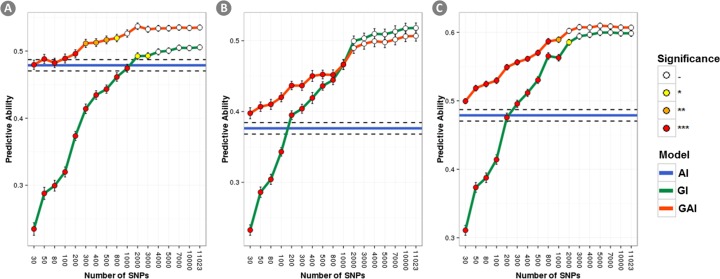
Accuracy of r_GP_ as a function of the number of randomly selected SNPs used to compute the GRM. Results for the three traits under analysis are reported: a) Yield; b) Lodging; c) Starch content. Accuracies obtained with the A+I, G+I and A+G+I, models are represented in blue, green and red, respectively. The color of the dots show if each r_GP_ was significantly lower than the highest observed r_GP_ obtained with each model.

Fig 3 shows the r_GP_ obtained when the number of SNPs was increased and the SNPs selection was random. For model GI rather low r_GP_ but significantly different from zero were observed even when a modest number of SNPs were used. Increasing the number of SNPs included in the analysis resulted in a sharp increase in r_GP_. However, around 2K SNPs were enough to reach the highest r_GP_ observed. Adding the **A** matrix to the model strongly reduced the loss of r_GP_ when few SNPs were used to compute the **G** matrix. In this scenario the maximum r_GP_ is reached with less SNPs (between 800–1000).

**Fig 4:**
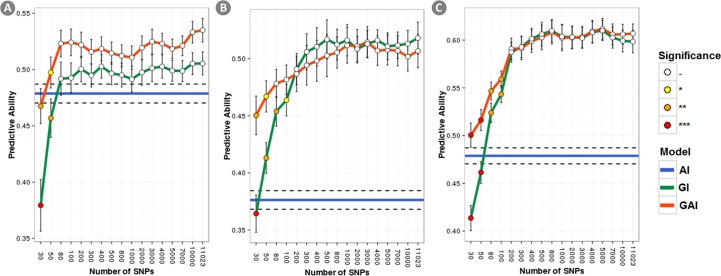
Accuracy of r_GP_ as a function of the number of GWAS-based selected SNPs used to compute the GRM. Results for the three traits under analysis are reported: a) yield; b) lodging; c) starch content. Accuracies obtained with the A+I, G+I and A+G+I, models are represented in blue, green and red, respectively. The color of the dots show if each r_GP_ was significantly lower than the highest observed r_GP_ obtained with each model.

**Fig 5:**
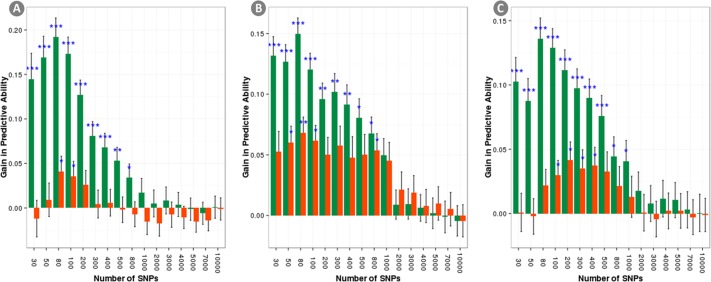
Gain in r_GP_ obtained by using a GWAS based marker selection instead than a random one. Results for the three traits under analysis are reported: a) yield; b) lodging; c) starch content. Stars showing the significance of the improvement are displayed.

Fig 4 shows the r_GP_ obtained when the number of SNPs was progressively increased and the SNPs were selected using the GWAS output. In this scenario, the maximum r_GP_ for each model were reached with very few SNPs (between 80 and 200) and, as before, the model GAI was outperforming the model GI reaching the highest r_GP_ at very low number of SNPs. Fig 5 shows a comparison between the r_GP_ for analysis using random SNPs selection and using GWAS-based SNPs selection. When few SNPs were used (less than 1000) the GWAS based selection always outperforms the random selection for the model GI. For the model GAI, the presence of **A** mitigate the r_GP_ difference among the two selection methods which were non-significant at very low number of SNPs when the prediction mostly rely on the pedigree. However, the GWAS-based SNPs selection still outperforms the random selection for SNPs size sets between ~100 and ~1K SNPs.

## Discussion

A large panel of commercial wheat breeding lines phenotyped for yield (Y), lodging (L) and starch content (SC) and genotyped with 15k SNPs chip was considered in this study. Variance components and heritabilities were estimated for these traits and the accuracies of the genomic predictions were investigated. The effect of training population size on r_GP_ was evaluated as well as the effect of reducing the number of markers used in the analysis either randomly or on the basis of GWAS results. For these different scenarios, models using pedigree records, genomic data or a combination of the two were compared.

### Variance components

Additive genetic relationship matrices describing inbreeding coefficient and coefficient of relationship between individuals have been extensively used in mixed model to estimate the additive genetic variance components of phenotypes under study [30]. As showed by our results, using simplified models assuming no relationship between individuals (model I, Eq 1) leads to lower estimates of genetic variance. For these models, the covariance between individuals is ignored and, as a consequence, the effective number of independent records is larger than in the reality leading to biases due to incorrectly specified covariance structure.

To overcome this problem, matrices of relationships among individuals are used. These relationship matrices can be calculated by using the pedigree information (**A**) or genomic markers information (**G**). Our results showed how the additive genetic variance traced by **A** was always higher than the one traced by **G**, and for two phenotypes (yield and starch content) it was equal to the total genetic variance. The pedigree-derived relationship matrices are expectation of the proportion of genome shared by individuals due to inheritance by descend. As a consequence, **A** represents unbiased estimation of the relationship matrices at the QTLs that affect the phenotype under study and give unbiased estimation of genetic variance [31]. Differently, **G** is an estimate of the realize proportion of genome shared by individuals. Such an estimate can be bias downwards due to absence or to imperfect LD between markers and causative QTLs leading to underestimation of genetic variances [32]. In agreement with literature [33,34], the LD in our wheat panel of commercial lines was extensive. Nevertheless, our results show that the SNPs set used was not sufficient to trace all the genetic variance. This can be a consequence of: i) uneven coverage of the genome; ii) systemic difference between the markers and the causative mutation (e.g. different allele frequency); iii) absence of markers specific of the population under analysis in the SNPs set used. This result indicates that, even in species with extended LD, precise estimation of genetic variance components would require a large and well distributed set of markers, including allele variants specific of the population under study. As an alternative we suggest to utilize both the genomic and the pedigree-based relationship matrices to estimate the genetic variance components.

### Genomic prediction accuracy

The GP accuracies yielded by models using **G** (when all the markers were considered) were always outperforming the one yielded by the models using **A**. This was consistent with other studies [31,35] and reflected the difference between **A** and **G**. While **A** indicates the expected fraction of genes identical by descent, **G** estimates the actual fraction of DNA shared by individuals or, in other words, it accounts for the specific Mendelian segregations. The advantage of using genomic data for making predictions is clear for cereal breeding, where large part of selection is made within advanced populations of full sibs. In this population BV estimate by only using the pedigree information would be the same for all the individuals. When all the SNPs were included in the analysis, the advantage of considering pedigree and markers jointly was small. This might be a consequence of redundancy between pedigree and markers information [36]. This result showed that even though the markers set under use might not trace all of the genetic variance, the pedigree contribution to a precise BV estimation was limited. As discussed later, this might not be the case when the markers number is reduced.

### Training population size

The choice of the training population is one of the most important elements determining the accuracy of the genomic prediction. Generally, TP with a large number of individuals, highly related to the BP are the one giving the most accurate GP [19]. Since one of the aims of GP is reducing the need of phenotyping, we explored how large the TP set (which need to have both markers and phenotypic scores) should be to yield useful prediction accuracies. By increasing the size of randomly chosen TP, we observed an increased r_GP_ until the accuracy plateaued ~700 lines. Several works in literature explored the effect of the TP size [18,19,24]. A general conclusion was that TP including hundred full sibs or few hundreds of half-sibs of the BP are sufficient to yield reasonably high GP accuracy, while thousands of far related individuals are needed to obtain similar results [14].

The lines considered in the present study were generated in three consecutive breeding cycles. Lines coming from the same breeding cycle were highly related to each other (full sib, and half sibs), while they were less related with lines coming from different breeding cycles. This represents the classical situation in wheat breeding schemes, where multiple crosses of few parents are used each year to generate the advanced lines which will undergo the final selection. Out of the ~700 lines need to reach the maximum r_GP_, on average a third were coming from the same breeding cycle of the line included in the BP. In accordance with the literature, we concluded that in standard breeding procedure, only few hundreds of the newly produced breeding lines need to be phenotyped while breeding values can be estimated through GP for a large number of the remaining ones at the only cost of genotyping.

The reported results also showed that including pedigree-based relationship matrices in the models do not mitigate the loss in information due a reduction of the TS size. However, a unified, so called ‘single step’, approach has been proposed in which phenotyped individuals with no genomic information available are jointly used with both genotyped and phenotyped individuals by using pedigree information [37,38]. Even though this scenario was not explored in the present work (because the aim was to investigate the possibility of reducing the need of phenotyping rather that the need of genotyping), it is important to consider that precise pedigree information can be used to increase the TP size without the need of extra genotyping.

### Linkage disequilibrium and GWAS

The LD between markers is a consequence of physical linkage and population structure [39]. Our result showed a moderate level of LD caused by population structure. This reflected the absence of a clear subdivision in genetically different groups among the considered breeding population. In accordance with other studies on commercial lines [33,40], the LD decay rate of physically linked markers was low in our population. Likewise, our analysis confirmed previously LD patterns in the wheat genomes [33,34] with the D genome showing a slower decay when compared to the A and the B genomes. The power to detect QTL is a function of mapping population size, marker density, extent of LD, and QTLs effects (Long and Langley, 1999). When the LD is widely extended, fewer markers are needed to detect markers/QTLs associations; similarly QTLs with large effect can be detected using smaller mapping populations [41]. The GWAS was able to identify few markers/QTLs association for each trait under study. However, the fraction of genomic variance explained by the associated markers ranged between 2% and 5% and the cumulative variance explained by all significant QTLs per trait was never higher than 10%. This lack of explained phenotypic variance is commonly observed in mapping studies [32]; given our experiment setup, we can identify three main reasons to explain this observation.

Firstly, the markers were not evenly distributed across the chromosomes with the D genome being under-represented. The chip used to genotype the population yielded 9,290 mapped SNPs functional for the GWAS, of which only 11.7% were located on D chromosomes. Even though this might have hampered QTL discovery in these regions, the D genome shows a reduced diversity compared to A and B [42], hence less genetic variation influencing the traits are expected to be found on the D genome.

Secondly, the marker set used was developed by genotyping a large germplasm panel with the aim of representing the global wheat genetic variation [33]. However, such SNPs chips are generally biased toward the particular panels of germplasm used to identify markers variants [20]. As a consequence, SNPs specific of the population under study might not be represent in the chip limiting the power to detect population specific QTLs. One possibility to circumvent these limitations is offered by the next generation sequencing (NGS) technologies which allow shifting from array-based genotyping assays based on a pre-defined SNPs panel to the direct sequencing of the populations of interest.

Finally, although a large population (~1K, lines) with several phenotypic observations per line (the overall number records per trait was ranging between 8k and 10k) was used, large part of the QTLs might have had a low effect limiting the GWAS power to identify significant associations. To support this idea, we consider a simplistic power calculation for our association study (following the methods presented by Feng et al. [43]). Given our populations size and assuming an LD between SNPs and QTLs of 0.5, the power of detecting locus with an effect equal to 5% was equal to 74% but it drastically dropped down to 1.8% for effect equal to 1%. This indicates that a large fraction of small effect QTLs could not be detected by our study. Giving the small fraction of the cumulative genetic variance explained by the associate markers we can conclude that the trait under study showed a complex genetic architecture governed by a large number of QTLs each with small effect [3].

### Reduction of marker sets

One of the main goals of our work was to test the possibility of reducing the markers needed for GP. Reducing the number of markers without decreasing the accuracy of the GP would allow increasing the number of genotyped lines (keeping the genotyping budget fixed). The expected genetic gain of a GP based selection is a function of the additive genetic variance observed for the trait, the square root of the prediction accuracy and the selection intensity. Increasing the size of the genotyped individual in the BP would result in an increased selection intensity and, ultimately in an increased genetic gain.

Our result showed that around 1k of randomly selected, spaced markers were enough to reach the maximum r_GP_ yielded by the full SNPs set. This result was in accordance with the findings of Spindel et al.[44] showing that using few thousand, evenly spaced, markers in a panel of *Oryza sativa* varieties was enough to maximize the observed r_GP_. It has been shown that, as long as at least one marker is in LD with each causative QTLs, the number of total markers used for GP as a little effect on the accuracy [45]. Given the slow decay of the LD observed in the population under study it was not surprising that around 10% randomly selected markers were sufficient to achieve the same r_GP_ yielded by the full SNPs set.

However, this observation did not mean that using a larger marker set would not yield higher r_GP_ but, rather, it was an indication that the density of the markers could be reduced with a limited effect on accuracy. As discussed before, the set of SNPs used was not able to trace all the genetic variance; including more SNPs located in uncovered regions of the genome might allow better tracing of all causative QTLs and, as a consequence, lead to increase r_GP_. This observation should encourage the utilization of genotyping assays using a moderate number of well distributed markers for GP in wheat.

As previously discussed, several factors contributed to limiting the possibility of detecting significant associations in the GWAS. However, this method allows ranking markers according to their genetic effect. GP performed by using subset of the total markers based on the GWAS ranking showed that the maximum r_GP_ could be achieve by using few hundreds markers (between 100 to 300 depending on the trait). Interestingly, a large part of these markers, which contributed to increase r_GP_, did not pass the significant threshold in the association analysis. This result might indicate that although the GWAS leads to few significant associations, the ranking of the whole markers (including the non-significant one) is meaningful and can trace the multiplicity of QTLs underling complex phenotypic traits. The traditional concept of MAS is based on the idea that few markers, strongly associated with the QTLs, can be identified and used to predict the performances of the breeding populations [4]. Here we propose a different idea, in which a larger number of markers (which is still a small fraction of the initial set), including some which are weakly associated with the phenotype, are selected after a testing their association to the phenotype in the TS. This selected SNPs panel is subsequently used to genotype the BP to make predictions of BV. This approach might extend the use of MAS to trait with a complex genetic architecture. However, two main observations need to be made. Firstly, new marker selection might be needed at each breeding cycle. Being the selected markers weakly associated with the phenotype their profile is expected to change considerably when different population are considered. Under the assumption that the TP is constantly updated to be closely related with the breeding population (as suggested by several authors e.g. Heffner et al. [46]) we suggest to: i) genotype the TP with a medium high number of well distributed markers, possibly deriving from direct sequencing of the population under study; ii) train the GP model and identify a subset of markers yielding the highest prediction; iii) genotype the BP with the selected markers and make prediction of their BV.

Secondly, using a reduced, selected set of markers would have a practical use primarily if it allows increasing the BP size without increasing the cost. Currently, few genotyping platforms allow scanning few selected markers on a set of individuals (as briefly discussed in the introduction). These methods allow cheap genotyping when few samples (in the magnitude of the hundreds) are analyzed but they are quickly outperformed by NGS-based assays when the number of genotyped individuals increases to some thousands. Given the current technology using GBS-based platforms to generate low coverage genotyping of unselected SNPs appear to be the most convenient approach; nevertheless changing in the available technology might change the future scenarios.

Finally, we showed how adding a pedigree-based relationship matrix in the prediction model can greatly mitigate the loss in predictive ability associated with a reduction of the number genomic markers both in a random and in a GWAS-based selection scenario. It is reasonable to expect that, as the number of molecular markers decreases, the relative contribution of pedigree information will increase and becomes highly relevant when very few markers are used to make prediction. If future GP strategies for wheat breeding will aim to reduce the number of genotyped markers to increase the BP size, keeping track of each line pedigree and using this information in the prediction model would be highly recommended.

## Conclusion

Our findings were based on a study of commercial advanced wheat breeding lines and corroborated the general conclusion that GP can be successfully implemented in practical breeding. Promising accuracies of genomic prediction were obtained by using 15k SNPs chip genotyping platform. However, we showed that markers yielded by such an assay were not sufficient to capture all of the genetic variance, concluding that a wider coverage of the genome might increase the r_GP_. The effect of the training population size was explored. Some hundred individuals closely related to the breeding population were found to be able to yield the highest prediction accuracies. This result indicates that whenever new advanced breeding lines are produced in a breeding cycle, it is possible to phenotype only few hundreds of them and generate prediction of the others by means of GP. This strategy can be very advantageous, especially for traits which require laborious and expensive phenotyping.

A GWAS study was able to identify few markers associated with the traits under study. However, the genetic variance explained by the associated markers was generally moderate, reinforcing the idea that a marker assisted selection strategy using few markers can be difficult to implement for complex traits as the ones considered in this study.

Moreover, we explored the effect of reducing the number of the markers used in the GP. Firstly, we selected the markers randomly showing that the maximum r_GP_ could be achieved with using around ten time less markers than the complete set. This observation led to the conclusion the marker density could be greatly reduced without affecting the r_GP_. In a second test, we reduced the markers number using the GWAS result as a selection criterion. This selection allow to reach maximum genomic prediction accuracies with few hundreds markers. This result proved that mapping approaches can be used to reduce the genotyping effort needed. Reducing the quantity of markers needed for GP can allow increasing the number of individual genotyped (by keeping the genotyping budget constant). This would result in increased selection intensity and ultimately in higher genetic gain.

Finally, the usefulness of using pedigree information was shown. Pedigree-based relationship matrix allowed more accurate estimate of the genetic parameters and greatly increased the r_GP_ when small marker sets were adopted. Pedigree records can be maintained at very low cost and should be automatically generated in any well managed breeding program. Nevertheless the breeding activity, and in particular the genomic selection, can greatly benefit from using this information.

## Supporting Information

S1 Table SNPs showing a significant association with the three traits under analysis. (XLSX)

S1 Fig Observed linkage disequilibrium. a) All wheat chromosomes combined; b) chromosome set A; c) chromosome set B d) chromosome set D.
(TIF)

S2 Fig Example of the GWAS based marker selection for lodging. a) top 30 markers selected; B) top 200 marker selected.
(TIF)
