# Supplementary figures and images for: Optimizing Training Population Size and Genotyping Strategy for Genomic Prediction Using Association Study Results and Pedigree Information. A Case of Study in Advanced Wheat Breeding Lines
Source: PLoS One. 2017 Jan 12;12(1):e0169606. doi: 10.1371/journal.pone.0169606 (PMC5231327; doi:10.1371/journal.pone.0169606)

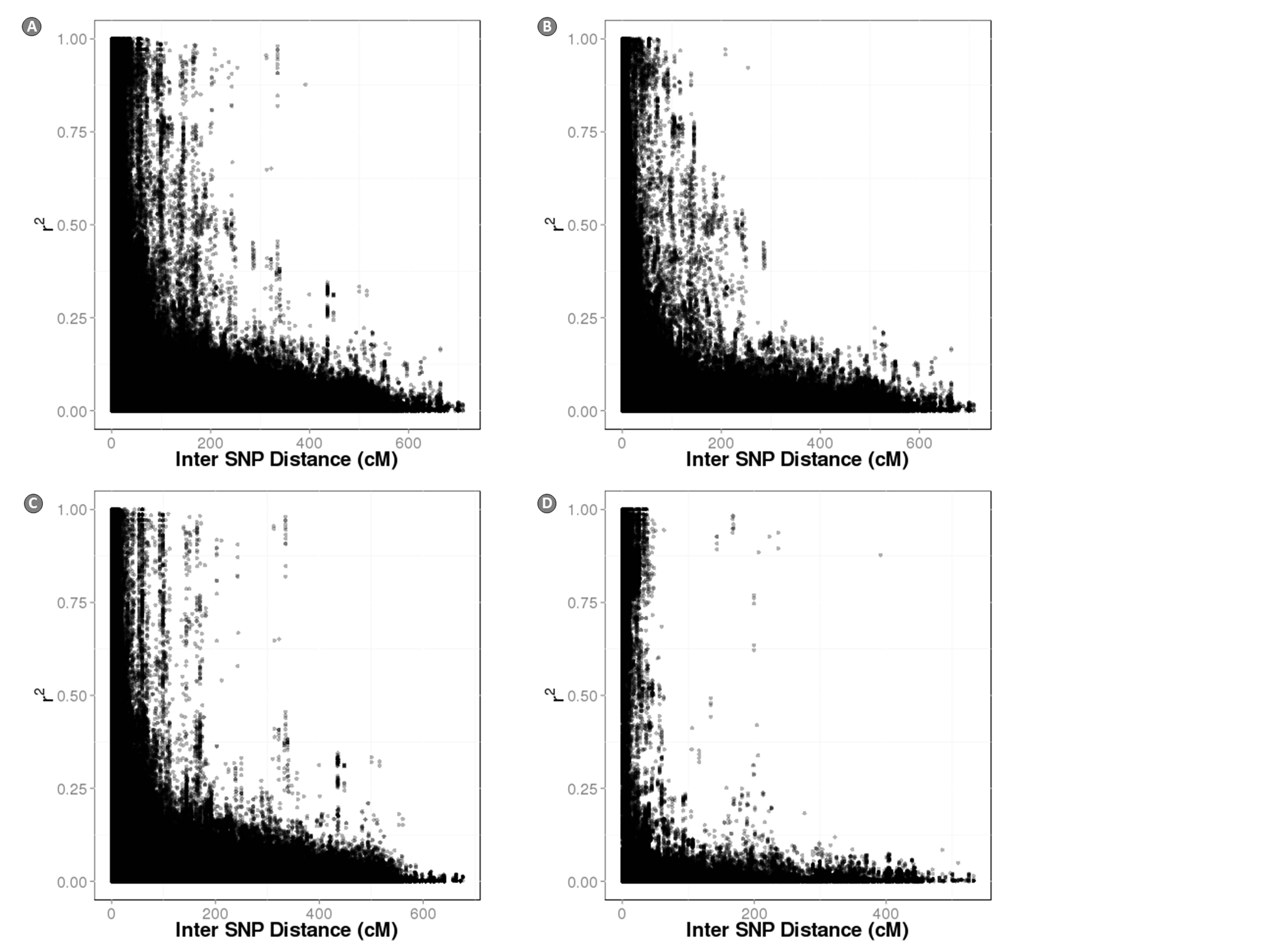

Supplement: S1 Fig — a) All wheat chromosomes combined; b) chromosome set A; c) chromosome set B d) chromosome set D. (TIF) [file pone.0169606.s002.TIF]

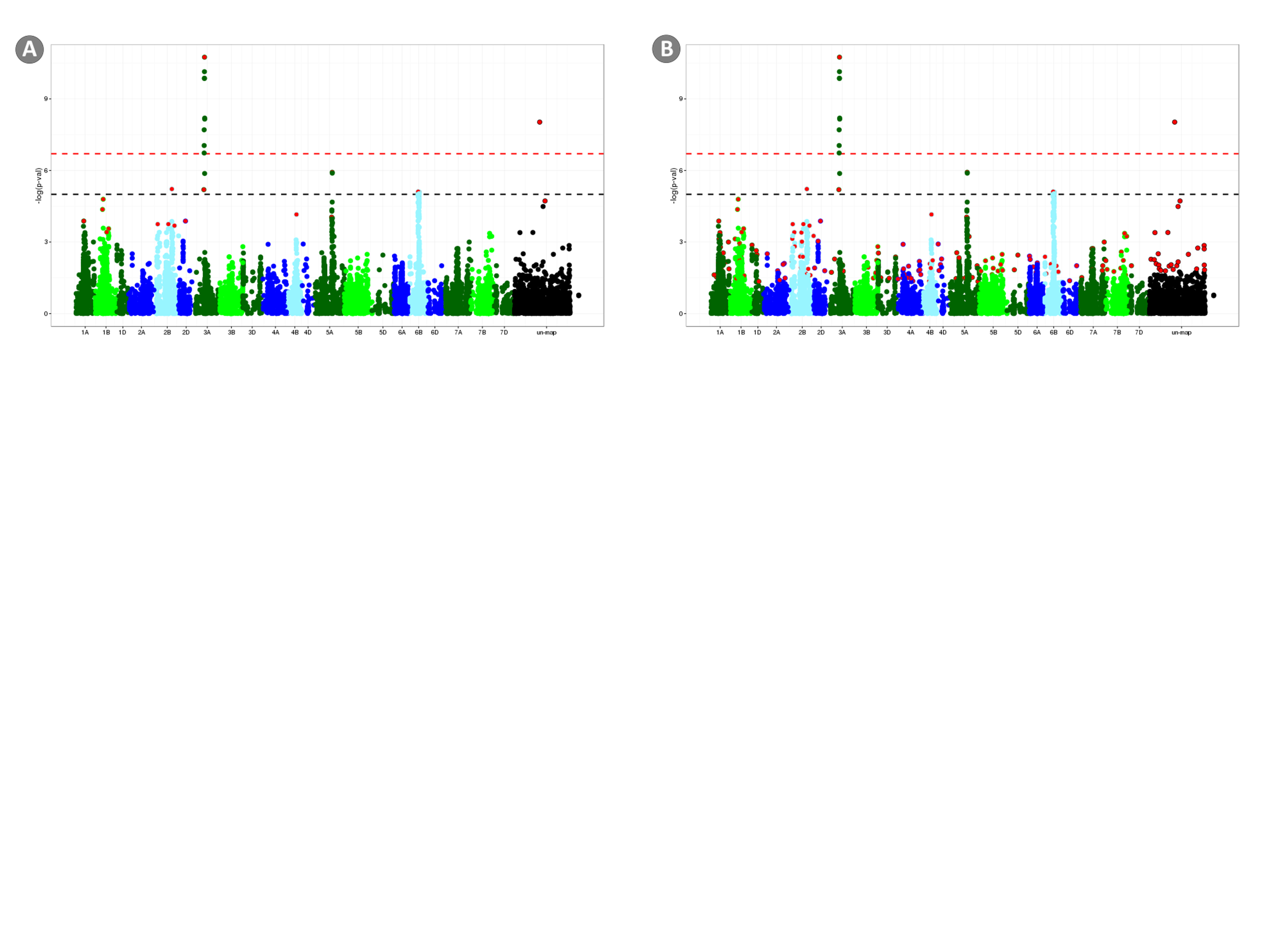

Supplement: S2 Fig — a) top 30 markers selected; B) top 200 marker selected. (TIF) [file pone.0169606.s003.TIF]
